# Association between migraine and epilepsy: a meta-analysis

**DOI:** 10.3389/fneur.2023.1276663

**Published:** 2024-01-05

**Authors:** Xiaohui Wu, Jiaxin Zhuang

**Affiliations:** Department of Neurology, Quanzhou Children's Hospital, Quanzhou, Fujian, China

**Keywords:** epilepsy, migraine, meta-analysis, association, systematic review

## Abstract

**Background:**

Epidemiological studies have demonstrated a comorbid association between migraine and epilepsy. However, despite the long history of this association, the exact nature of the relationship between migraine and epilepsy remains largely unresolved. Therefore, it is crucial to conduct a meta-analysis in order to thoroughly investigate the relationship between migraine and epilepsy.

**Methods:**

Odds ratios (ORs) or relative risks (RRs) and 95% confidence intervals (CIs) regarding association between migraine and epilepsy were summarized using STATA 12.0 software.

**Results:**

There was an 80% increase in the lifetime prevalence of migraine among patients with epilepsy, compared to those without epilepsy with a random effects model (OR/RR: 1.80, 95% CI: 1.35 to 2.40, I^2^ = 97.5%, *p* < 0.001). There was an 80% increase in the lifetime prevalence of epilepsy among patients with migraine, compared to those without migraine with a random effects model (OR/RR: 1.80, 95% CI: 1.43 to 2.25, I^2^ = 80.6%, *p* < 0.001).

**Conclusions:**

It is important to note the comorbid association between migraine and epilepsy examined in the study.

## 1 Introduction

Migraine and epilepsy are two frequently seen neurological disorders, characterized by paroxysmal clinical manifestations (1). Despite the commonly accepted notion that migraine is primarily a neurological disorder with a strong genetic basis (2), it has been found to be frequently co-occurring with a wide array of other medical conditions. Numerous studies have identified a bidirectional relationship between migraine and various disorders, including those affecting the neurological, psychiatric, cardiovascular, cerebrovascular, gastrointestinal, metaboloendocrine, and immunological systems (3). Furthermore, epilepsy, being a complex disorder, is often accompanied by a range of comorbidities. Conditions such as depression, anxiety, dementia, migraine, heart disease, peptic ulcers, and arthritis are up to eight times more prevalent among individuals with epilepsy compared to the general population (4). Since the late nineteenth century, John Hughlings Jackson's observation on the coexistence of migraine and epilepsy has been acknowledged (5). In addition, risk factors, such as positive family history and comorbid affective disorders, along with triggering factors like alcohol consumption, menstruation, and irregular sleep patterns, are commonly observed in both migraine and epilepsy. Moreover, prophylactic medical agents, such as valproic acid and topiramate, also demonstrate similarities in their utilization in migraine and epilepsy. Epidemiological studies have demonstrated varying prevalence rates for migraine and epilepsy in different populations. The frequency of migraine has been reported to range from 5 to 18% in healthy individuals, while in epilepsy patients, the frequency ranges from 8 to 24% (6). Similarly, the prevalence of epilepsy in healthy populations has been reported to be between 0.5 and 1.5%, whereas in migraine patients, it ranges from 1 to 17% (7, 8).

However, despite the long history of this association, the exact nature of the relationship between migraine and epilepsy remains largely unresolved. Brodtkorb et al. (9) and Hesdorffer et al. (10) reported that there was insufficient evidence to establish a definitive correlation between migraine and epilepsy. Therefore, it is crucial to conduct a meta-analysis in order to thoroughly investigate the relationship between migraine and epilepsy.

## 2 Methods

The present article was performed according to the Preferred Reporting Items for Systematic reviews and Meta-Analysis (PRISMA) guideline (11).

### 2.1 Search strategy, inclusion criteria, and exclusion criteria

The article searched for literatures published before July 2023 in databases (PubMed, Web of Science, EMBASE, Medline and Google Scholar). We used these search terms: (“migraine” OR “headache”) AND (“epilepsy” OR “seizure” OR “falling sickness”).

The study included studies in accordance with these inclusion criteria as follows: (1) studies investigated migraine; (2) studies investigated epilepsy.

**Figure 1:**
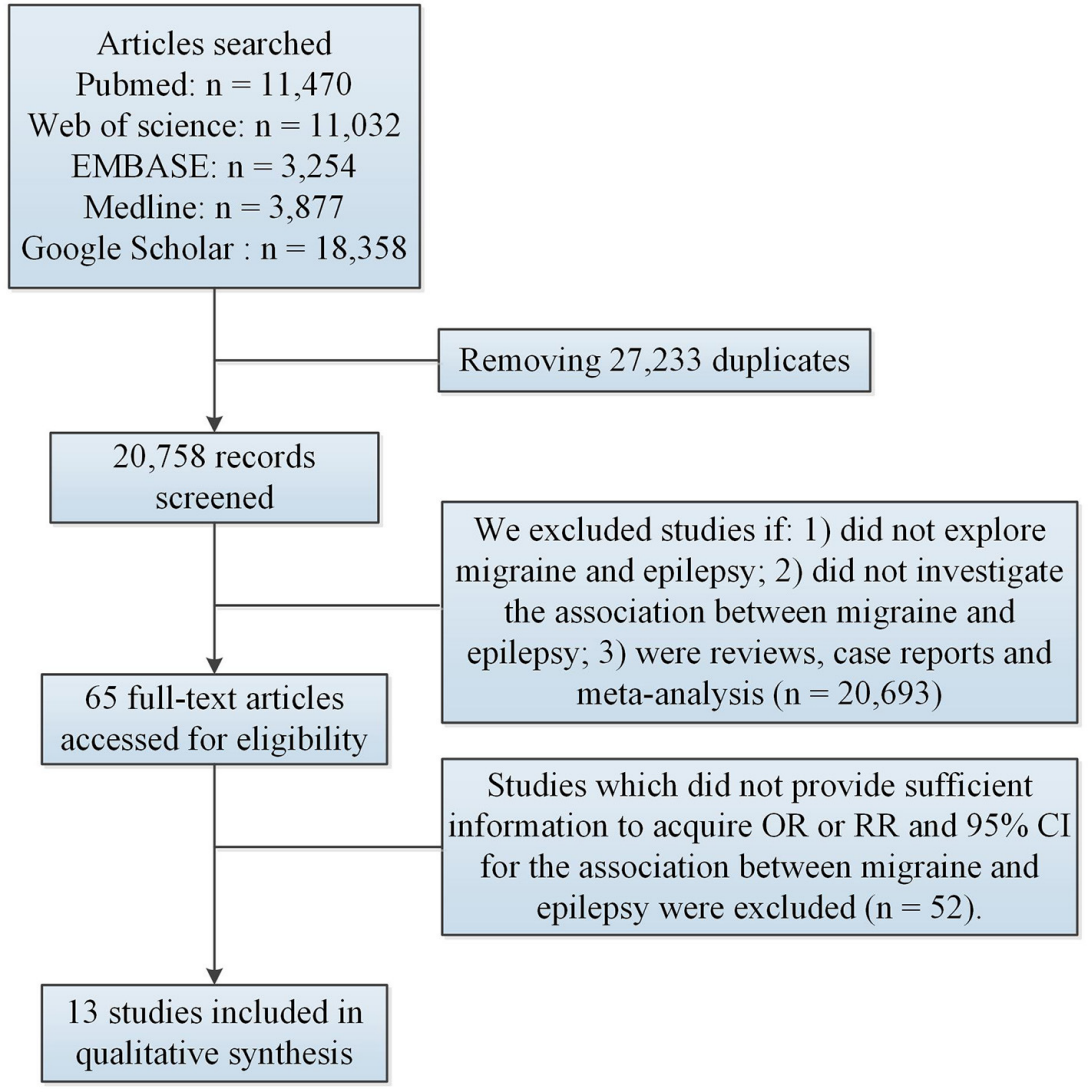
Flow of information through the different phases of meta-analysis. CI, confidence interval; OR, odds ratio; RR, relative risk.

We used these exclusion criteria: (1) studies which did not investigate the association between migraine and epilepsy; (2) studies which did not provide sufficient information to acquire odds ratio (OR) or relative risk (RR) and 95% confidence interval (CI) for the association between migraine and epilepsy; (3) reviews, case reports and meta-analysis. Figure 1 illustrated the flow chart of the study search and selection process.

### 2.2 Data extraction and meta-analysis

Two researchers extracted data from finally included studies. We extracted these data: Author and publication year, study location, study type, population, sample size, mean age, gender, epilepsy diagnosis, migraine diagnosis, adjusted variables and results.

ORs or RRs and 95% CIs regarding association between migraine and epilepsy were summarized using STATA 12.0 software. When *p*-value of *Q*-test was ≤ 0.05 and I^2^ was higher than or equal to 50%, a random effects model was used to compute all results with the high heterogeneity. When *p*-value of *Q*-test was higher than 0.05 and I^2^ was lower than 50%, a fixed effects model was used to summarize all results with the low heterogeneity. Subgroup studies (for different races, different study types, migraine diagnosed with different methods and adjusted or unadjusted effect estimates) were used to explore the source of heterogeneity. Meta-regression was used to investigate the source of heterogeneity. We used sensitivity analysis to evaluate stabilization of study. We used funnel plot, Begg's test and Egger's test to evaluate publication bias.

## 3 Results

### 3.1 Study characteristics

After removing duplications, 27,233 articles remained. Subsequently, 20,693 articles were excluded based on the screening of titles and abstracts. Among 20,693 articles, 18,630 did not explore migraine or epilepsy; 1,952 did not investigate the association between migraine and epilepsy; 46 were reviews, case reports and meta-analysis. Among 65 potentially relevant articles, 52 were excluded because they did not provide sufficient information to acquire OR or RR and 95% CI for the association between migraine and epilepsy. Ultimately, 13 studies (9, 10, 12–22) met the eligibility criteria and were eligible for the comprehensive analysis. Table 1 illustrated study characteristics. These studies investigated the association between epilepsy and the risk of migraine. Among the 13 studies, six studies (9, 13, 16, 17, 20, 22) (including N = 3,863,625 individuals) explored the association between migraine and the risk of epilepsy.

**Table 1:** Characteristics of included studies.

**References**	**Country**	**Study type**	**Population**	**Sample size**	**Mean age (years)**	**Gender (male%)**	**Epilepsy diagnosis**	**Migraine diagnosis**	**Adjustment**	**Result**
Jalava and Sillanpää (15)	Finland	Cohort	Hospital/clinic-based as well as national administrative database	267	35.6	NR	Clinical assessment by study neurologist	Structured interview by study neurologist	No	Prevalence of migraine in epilepsy 1.92 (0.89, 4.25)
Gaitatzis et al. (22)	UK	Cohort	211 participating general practices	1,041,643	≥16	48.9	Unvalidated ICD codes	Unvalidated ICD codes	No	Prevalence of migraine in epilepsy 1.60 (1.43, 1.79); Prevalence of epilepsy in migraine 1.63 (1.45, 1.84)
Téllez-Zenteno et al. (20)	Canada	Cohort	Nationwide cohort study via cluster sampling	49,026/130,822	30	49.0	Unvalidated telephonead ministered single questionnaire item	Unvalidated telephone-administered questionnaire	No	Prevalence of migraine in epilepsy Community Health Survey: 2.0 (1.7-2.3); National Population Health Survey: 2.6 (2.2–3.0); Prevalence of epilepsy in migraine Community Health Survey: 2.24 (1.86-2.70); National Population Health Survey: 3.17 (2.29–4.39)
Nuyen et al. (17)	Netherlands	Cross-sectional	134 participating general practitioners versus those without epilepsy or migraine	2,730,468	42.3	49.4	Unvalidated ICPC codes	Unvalidated ICPC codes	No	Prevalence of migraine in epilepsy 1.39 (0.76–2.54); Prevalence of epilepsy in migraine 1.51 (0.99, 2.33)
Hesdorffer et al. (10)	Iceland	Case-control	All individuals in the country with newly diagnosed epilepsy aged >10 years along with two age and sex matched controls	834	34	NR	Nationwide surveillance system to identify possible cases of epilepsy (those diagnosed by MD) which were then confirmed by review of medical records by study nurse	Unvalidated structured interview although unclear by whom	Age and gender	1.26 (0.91, 1.77)
Brodtkorb et al. (9)	Norway (Vaga)	Cohort	18–65-yearold inhabitants of the Vaga community	1,666	35	49.0	Unvalidated single questionnaire item administered by MD	Semi-structured headache interview administered by MD	No	Prevalence of migraine in epilepsy 0.93 (0.51, 1.65); Prevalence of migraine in epilepsy 0.65 (0.31, 1.36)
Le et al. (16)	Denmark	Cohort	All persons enrolled in the nationwide Danish Twin Registry	31,143	44–45	45.8	Unvalidated selfadministered single questionnaire item	Validated self-administered questionnaire (but with poor validity which was not used to correct the prevalence estimates)	No	Prevalence of migraine in epilepsy 1.53 (1.13–2.08); Prevalence of epilepsy in migraine 1.52 (1.27, 1.82)
Ottman et al. (18)	USA	Case–control	Persons with versus without epilepsy consisting of a nationwide random sample of one person ≥18 years old per household	6,976	NR	39.8	Validated self-administered single questionnaire item	Unvalidated selfadministered questionnaire	Sex, age, income, population density, census region, prior head injury, prior stroke, and survey panel	1.36 (1.25–1.48)
Baldin et al. (13)	Iceland (Reykjavik)	Cohort	All children attending grades 1–10 at almost all public and private schools in the administrative district	9,679	10.8	49.7	Unvalidated single questionnaire item self-administered by a parent	Unvalidated multi-item questionnaire algorithm self-administered by a parent	Age	Prevalence of migraine in epilepsy 2.02 (1.17–3.51); Prevalence of epilepsy in migraine 2.05 (1.18, 3.55)
Selassie et al. (19)	United States	cohort	The South Carolina (SC) statewide hospital discharge and emergency department (ED) visit datasets	64,188	41.6 ± 22.5	48.7%	PWE were defined based on at least one ICD-9-CM code for epilepsy	ICD-9-CM codes	All covariables	3.37 (3.23-−3.52)
Wang et al. (21)	China	Cross-sectional	Outpatient clinic of the Epilepsy Center of PLA General Hospital	1,109	28.2 (range 18–64)	54.7%	All the patients had a definite diagnosis of epilepsy as determined by at least two epileptologists.	A preictal headache was defined as a headache starting not more than 24 h prior to the seizure and lasting until the onset of a seizure.	No	1.43 (1.15, 1.77)
Dedei Daryan et al. (14)	Turkey	Cohort	Bakirkoy Prof. Dr. Mazhar Osman Training and Research Hospital for Psychiatric	200	27.89 ± 9.12	36.9%	ILAE 2010 criteria	International Classification of Headache Disorders criteria, III-beta	No	1.613 (0.835-3.236)
Agbetou et al. (12)	Benin	Cross-sectional	Neurology Department of the Teaching Hospital of Borgou	30/90	32 ± 15	59.2%	ILAE definition	The ICHD-3 beta criteria of 2013	Age, sex and living area	8.53 (2.6-28.0)

ICD, International Classification of Diseases; ICPC, International Classification of Primary Care; ILAE, International League Against Epilepsy; MD, medical doctor; NR, not reported; UK, United Kingdom; USA, United States.

### 3.2 Association between epilepsy and risk of migraine

**Figure 2:**
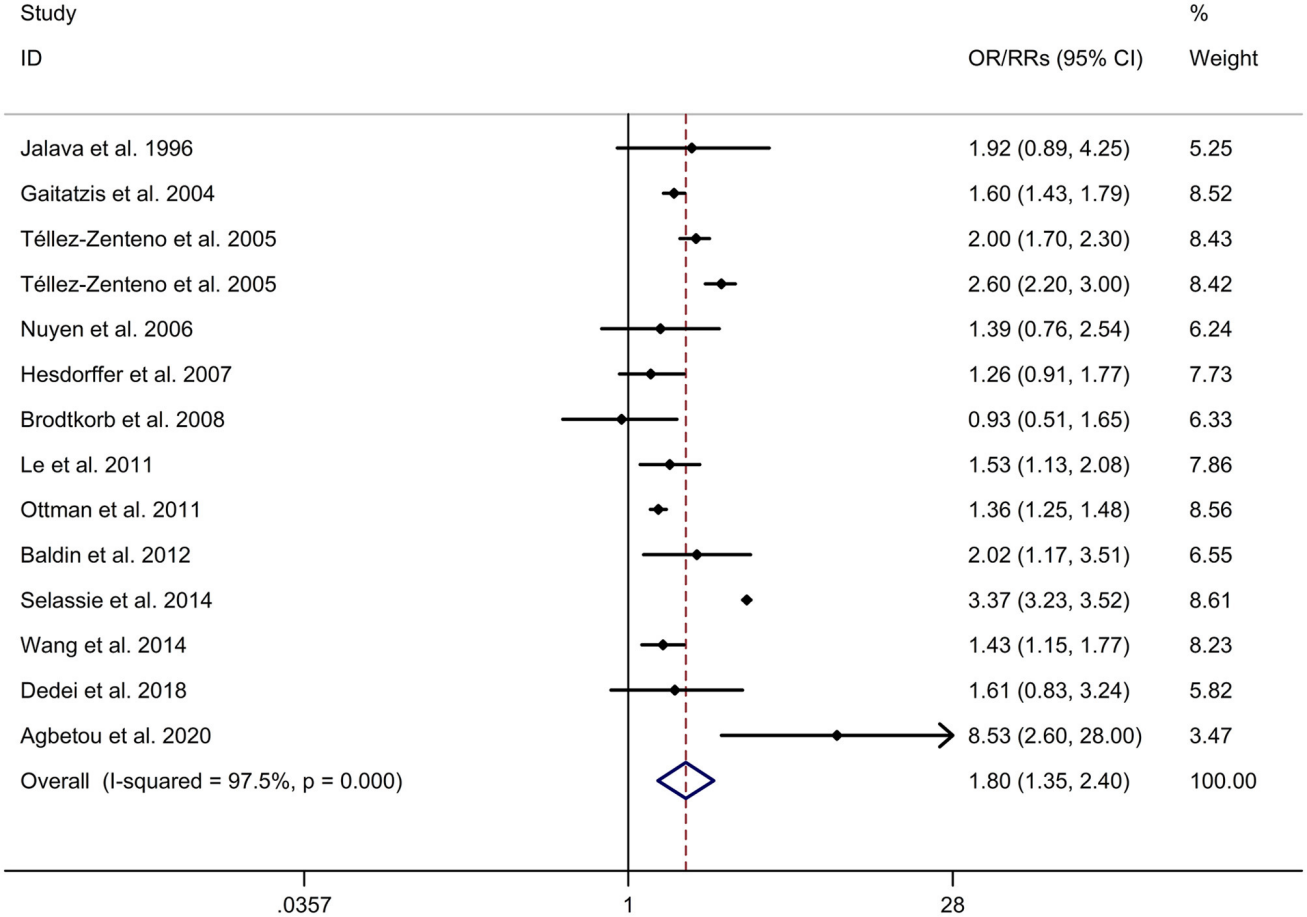
Forest plot for association between epilepsy and risk of migraine. CI, confidence interval; OR, odds ratio; RR, relative risk.

**Figure 3:**
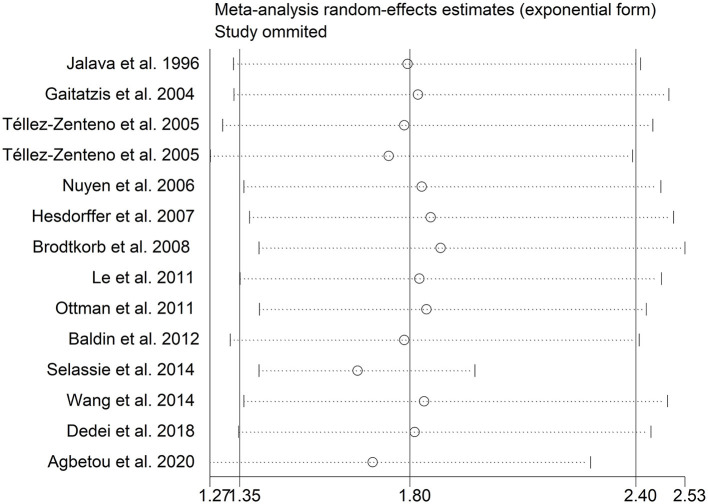
Sensitivity analysis for association between epilepsy and risk of migraine.

**Figure 4:**
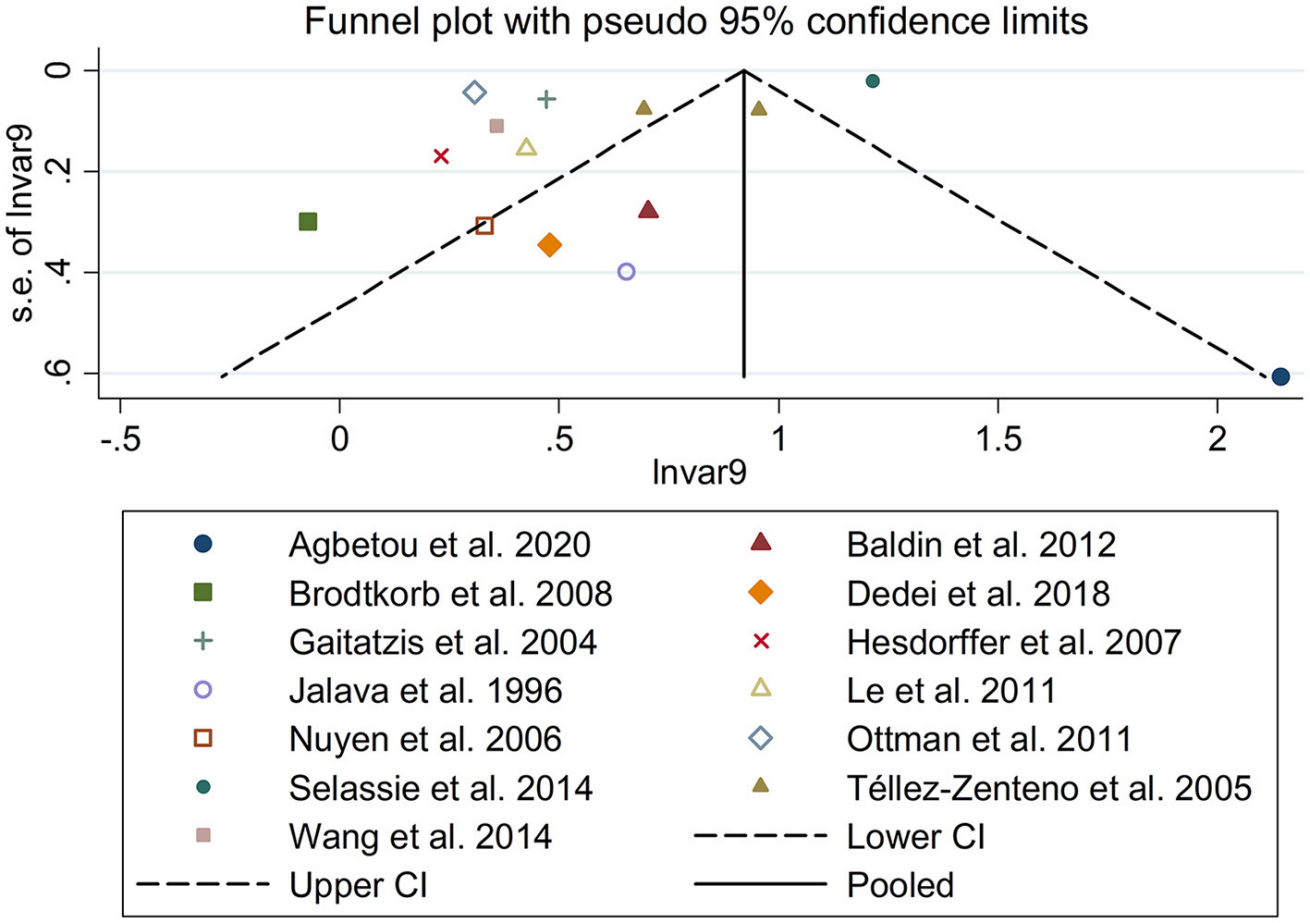
Funnel plot for association between epilepsy and risk of migraine.

There was an 80% increase in the lifetime prevalence of migraine among patients with epilepsy, compared to those without epilepsy with a random effects model (OR/RR: 1.80, 95% CI: 1.35 to 2.40, I^2^ = 97.5%, *p* < 0.001; Figure 2). Subgroup analysis reported a 73% increase in the lifetime prevalence of migraine among patients with epilepsy, compared to those without epilepsy among Caucasian populations (OR/RR: 1.73, 95% CI: 1.28 to 2.35; Supplementary Figure 1). Subgroup analysis reported a significant association between epilepsy and the risk of migraine in cohort and cross-sectional studies (cohort studies: RR: 1.91, 95% CI: 1.41 to 2.59; cross-sectional: OR: 2.03, 95% CI: 1.00 to 4.10; Supplementary Figure 2). In studies where epilepsy was identified by clinical assessment, the pooled OR/RR was 1.40 (95% CI: 1.17, 1.67), whereas the pooled was 2.28 (95% CI: 1.35, 3.84) and 1.71 (95% CI: 1.28, 2.28) when cases were identified using International Classification of Diseases (ICD)/International Classification of Primary Care (ICPC) codes or with a formal questionnaire (Supplementary Figure 3). The overall adjusted OR/RR was 2.18 (95% CI: 1.26, 3.79), whereas the overall unadjusted OR/RR was 1.68 (95% CI: 1.38, 2.05) (Supplementary Figure 4). Meta-regression study reported that publication year, age and gender were not responsible for the heterogeneity across included studies (publication year: *p* = 0.365; age: *p* = 0.918; gender: *p* = 0.361). Sensitivity analysis showed no change in direction of effect when any study was excluded from meta-analysis (Figure 3). Funnel plot, Begg's test and Egger's test showed no significant risk of publication bias (Figure 4; Begg's test: *p* = 0.090; Egger's test: *p* = 0.091).

### 3.3 Association between migraine and risk of epilepsy

**Figure 5:**
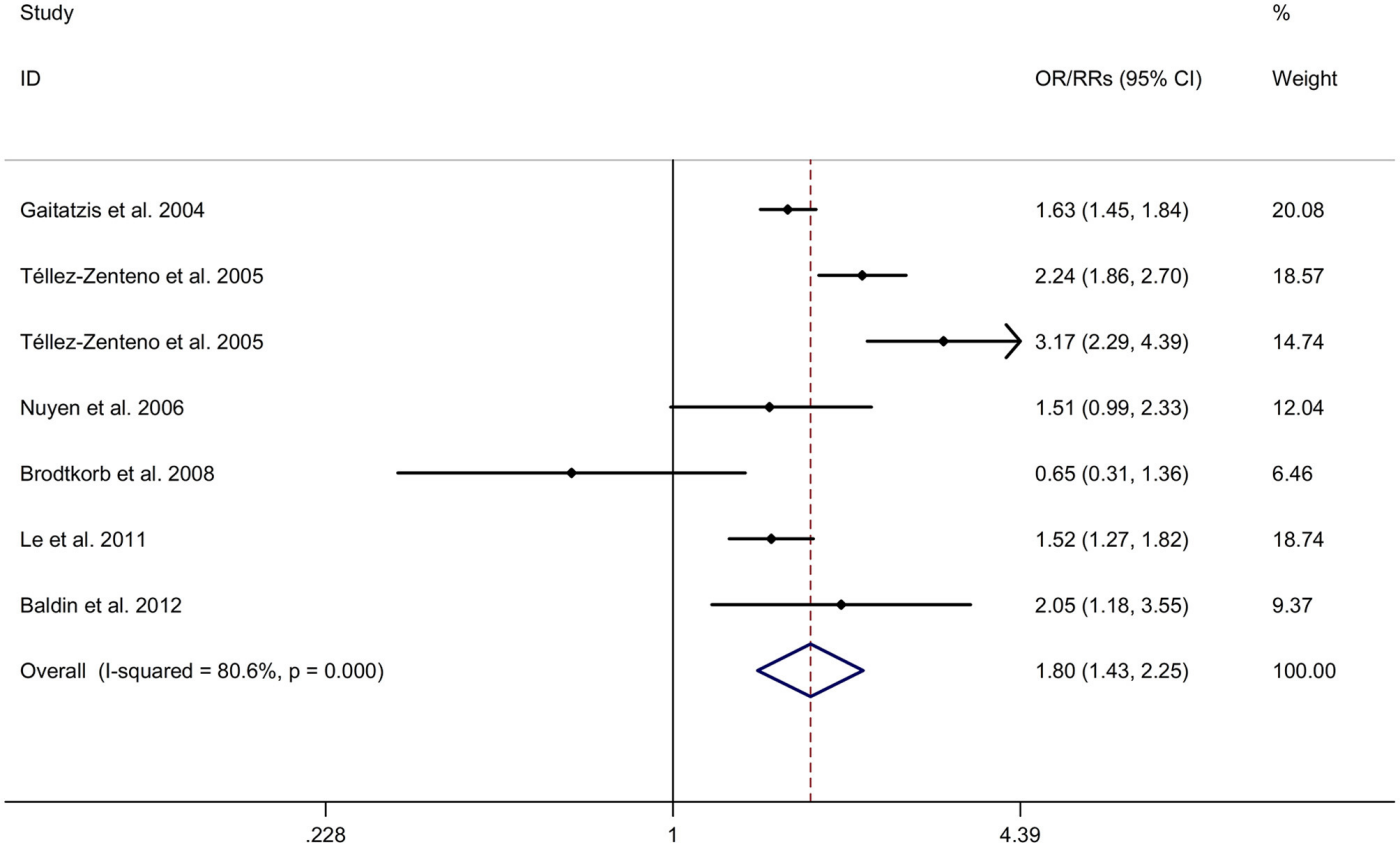
Forest plot for association between migraine and risk of epilepsy. CI, confidence interval; OR, odds ratio; RR, relative risk.

**Figure 6:**
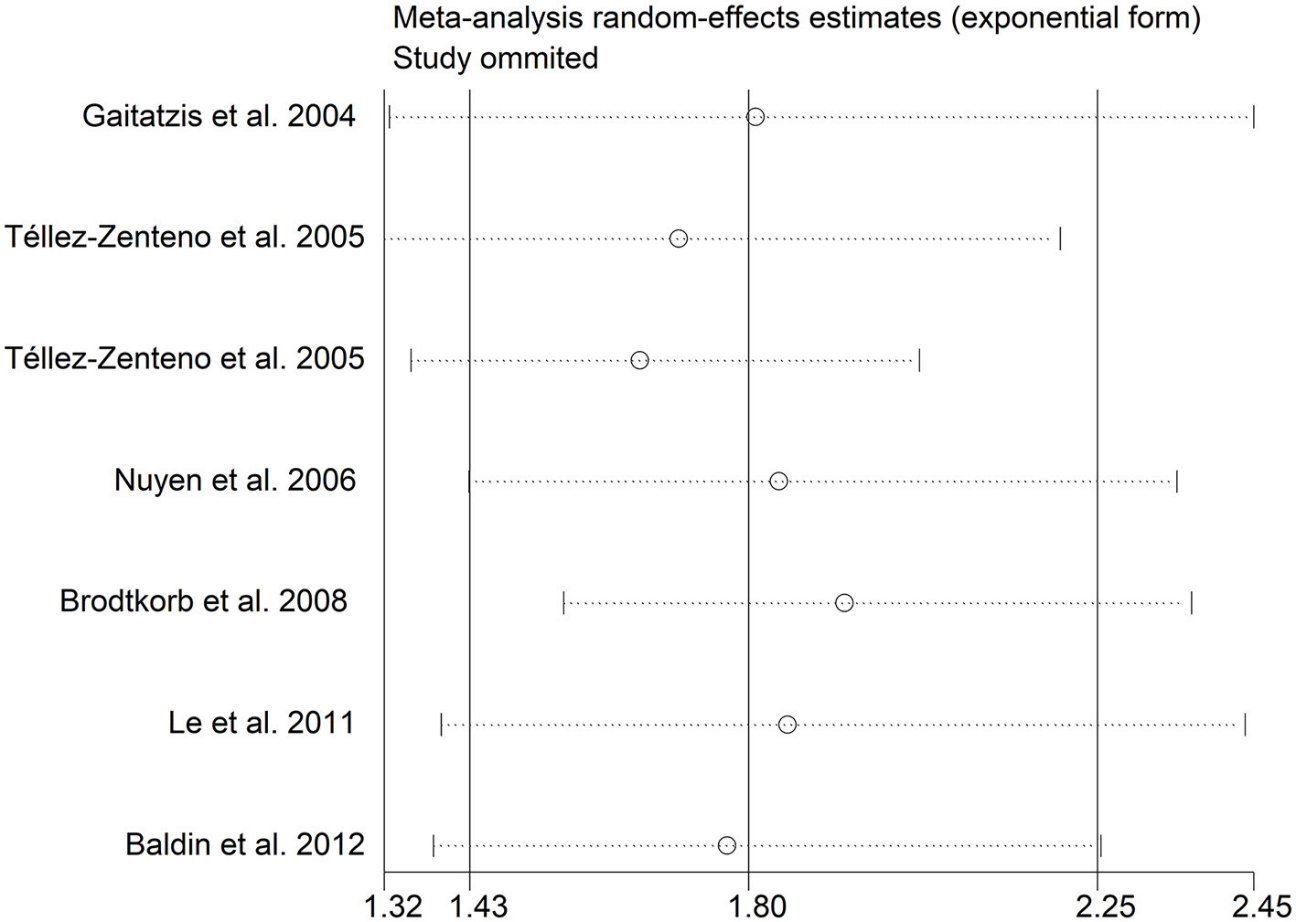
Sensitivity analysis for association between migraine and risk of epilepsy.

**Figure 7:**
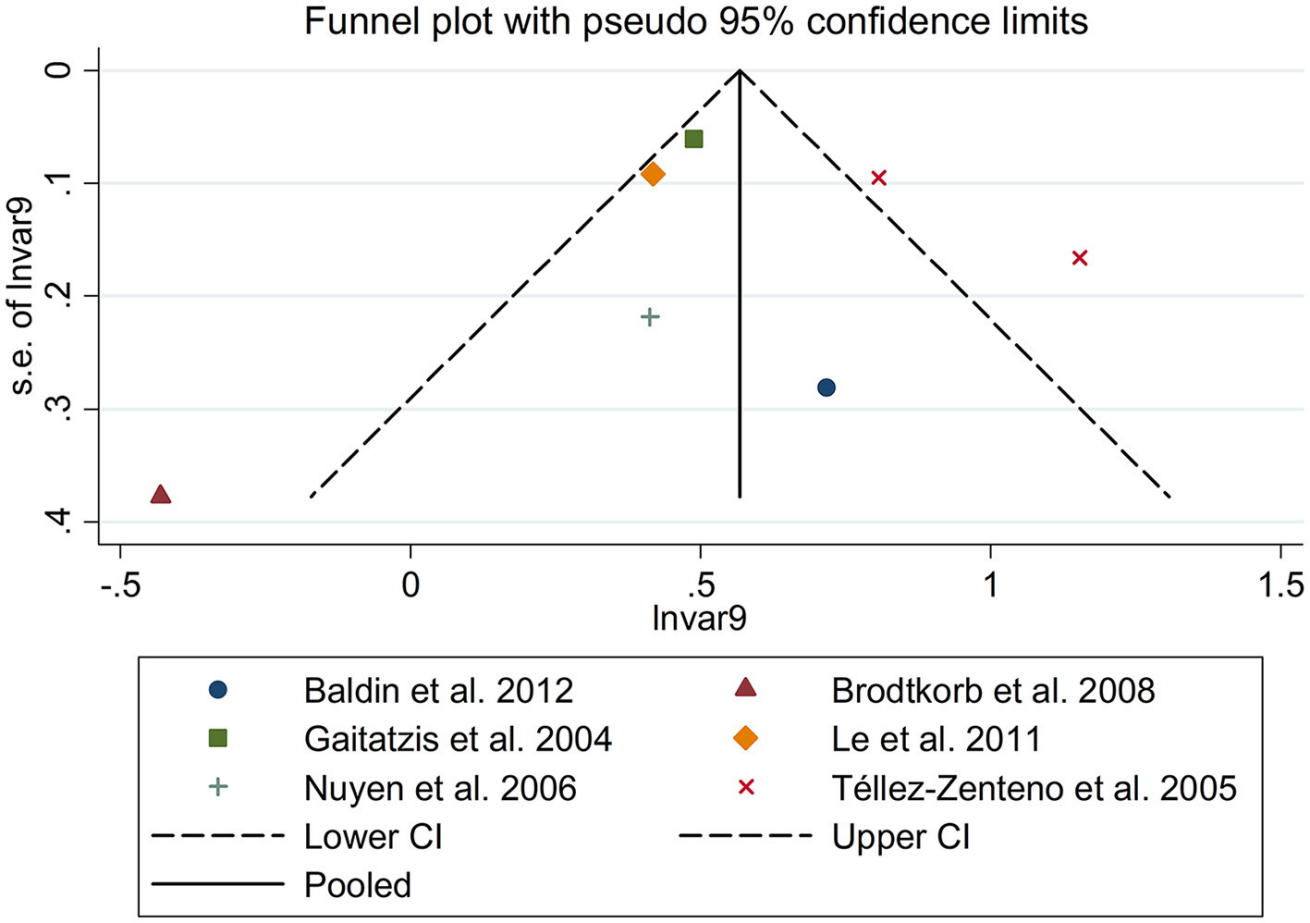
Funnel plot for association between migraine and risk of epilepsy.

There was an 80% increase in the lifetime prevalence of epilepsy among patients with migraine, compared to those without migraine with a random effects model (OR/RR: 1.80, 95% CI: 1.43 to 2.25, I^2^ = 80.6%, *p* < 0.001; Figure 5). Subgroup analysis reported an 84% increase in the lifetime prevalence of epilepsy among patients with migraine, compared to those without migraine in cohort studies (RR: 1.84, 95% CI: 1.43 to 2.35; Supplementary Figure 5). In studies where epilepsy was identified with a formal questionnaire, the pooled OR/RR was 1.86 (95% CI: 1.31, 2.64) (Supplementary Figure 6). The overall unadjusted OR/RR was 1.77 (95% CI: 1.39, 2.25) (Supplementary Figure 7). Meta-regression study reported that publication year, age and gender were not responsible for the heterogeneity across included studies (publication year: *p* = 0.295; age: *p* = 0.282; gender: *p* = 0.376). Sensitivity analysis showed no change in direction of effect when any study was excluded from meta-analysis (Figure 6). Funnel plot, Begg's test and Egger's test showed no significant risk of publication bias (Figure 7; Begg's test: *p* = 0.881; Egger's test: *p* = 0.938).

## 4 Discussion

In our study, migraine was more frequent in patients with epilepsy than in those without epilepsy. The result was consistent with a previous meta-analysis, which reported that there was an overall 52% increase in the prevalence of migraine among patients with epilepsy vs. those without epilepsy (23). In addition, it has been observed that the prevalence of epilepsy is higher among individuals with migraine, compared to those without migraine. The result was consistent with a previous meta-analysis (23), which reported that there was an overall 79% increase in the prevalence of epilepsy among migraineurs vs. those without migraine. Our literature search yielded a comprehensive selection of 13 studies involving a substantial cohort of 3,863,625 participants. This extensive pool of data enabled us to obtain more precise and potentially more accurate estimates of OR and RR compared to the individual primary studies. Additionally, it provided us with the opportunity to explore the potential factors contributing to any observed heterogeneity among these studies. This meta-analysis aims to examine the existing evidence concerning the comorbid association between migraine and epilepsy.

The understanding of the association between migraine and epilepsy remains incomplete due to a lack of fully elucidated underlying mechanisms. It is likely that there are common multifactorial mechanisms that contribute to this association. One possible explanation is neuronal hyperexcitability, which increases the risk of both diseases. This hyperexcitability can be caused by a higher concentration of extracellular glutamate, the main excitatory neurotransmitter, resulting in a cortical spreading depression (CSD) and convulsions (24–26). Migraine is characterized by an initial excess of neuronal activity, leading to CSD and aura, while epilepsy is characterized by neuronal over activity and rhythmic firing of neurons during a seizure (27). Migraine aura may act as a trigger for epileptic seizures (28), which is why some antiepileptic drugs are used in migraine prophylaxis. Channelopathies involving sodium and potassium ions, which can trigger CSD, may be a common pathogenic mechanism in migraine and epilepsy (26). Another focus of research is the hereditary link between epilepsy and migraine. A genetic link is particularly evident in familial hemiplegic migraine, a Mendelian transmission type of migraine. Mutations in genes such as ATP1A2, SCN1A, and CACNA1A, which are associated with familial hemiplegic migraine, have also been implicated in various types of epilepsy and febrile seizures (29, 30). However, the hypothesis of a clear common genetic susceptibility still needs further investigation due to the likely complex polygenic multifactorial inheritance and the role of gene-environment interactions (31). Finally, affective disorders can often accompany both migraine and epilepsy, suggesting a bidirectional relationship (32, 33). Studies have shown a correlation between migraine and depression, anxiety in epilepsy patients (32, 34). Interictal headache is specifically linked to depression, while postictal headache is related to depression and suicidality (12). In summary, the association between migraine and epilepsy is not fully understood, but it is likely that common multifactorial mechanisms, such as neuronal hyperexcitability and channelopathies, play a role. Hereditary factors and affective disorders may also contribute to this association. Further research is needed to fully elucidate the underlying mechanisms and develop targeted therapies for these diseases.

There are a few limitations in the study that should be acknowledged. Firstly, it was inevitable to encounter heterogeneity across the included studies. Although meta-regression and subgroup analyses were performed to identify potential sources of heterogeneity, the exact cause of heterogeneity remains unclear. Further investigation is needed to better understand this issue. Secondly, we could not ignore the possibility of an “overlap” in the rare case of “ictal epileptic headache” in whom an headache could be the sole ictal semeiological manifestations during the ictal phase of an epileptic seizure (35).

## 5 Conclusions

It is important to note the comorbid association between migraine and epilepsy examined in the study. This association not only has implications for understanding the relationship between the two conditions but also holds therapeutic significance. Certain antiepileptic drugs may have benefits for migraine prophylaxis, and additional considerations should be taken into account when treating individuals with both conditions. Further exploration is warranted to explore the potential of these treatments and their effectiveness in managing comorbid migraine and epilepsy.

## Data availability statement

The original contributions presented in the study are included in the article/Supplementary material, further inquiries can be directed to the corresponding author.

## Author contributions

XW: Formal analysis, Investigation, Methodology, Project administration, Software, Writing – original draft, Writing – review & editing. JZ: Methodology, Project administration, Software, Writing – original draft, Writing – review & editing.
