# Supplementary material for: Association between migraine and epilepsy: a meta-analysis
Source: Front Neurol. 2024 Jan 5;14:1276663. doi: 10.3389/fneur.2023.1276663 (PMC10796653; doi:10.3389/fneur.2023.1276663)
Supplement: Supplementary file 1 [file Data_Sheet_1.docx]

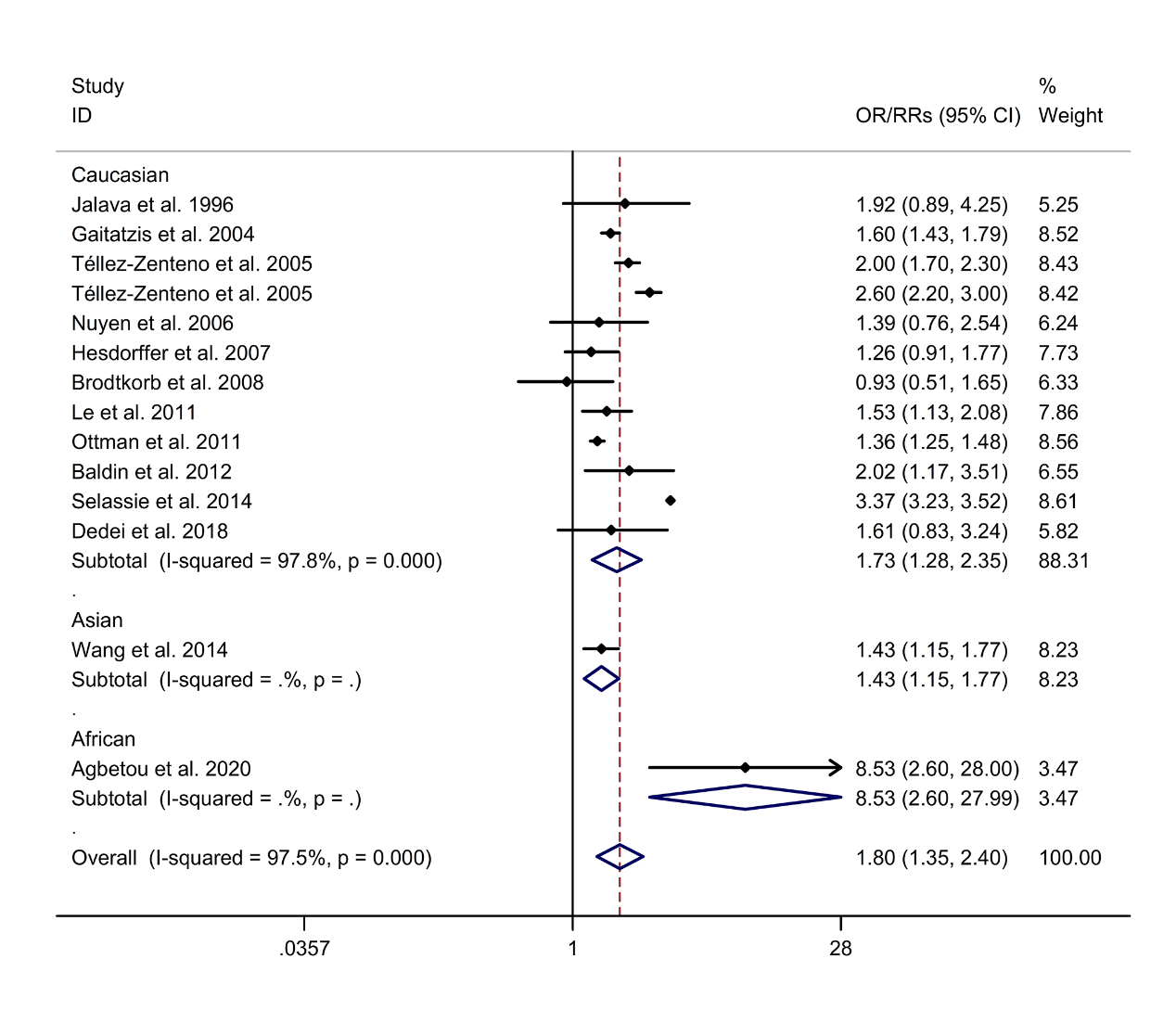


Supplementary figure 1. Subgroup analysis for association between epilepsy and risk of migraine among different [race](https://dict.youdao.com/w/race/#keyfrom=E2Ctranslation)s. Abbreviations: CI, confidence interval; OR, odds ratio; RR, relative risk.


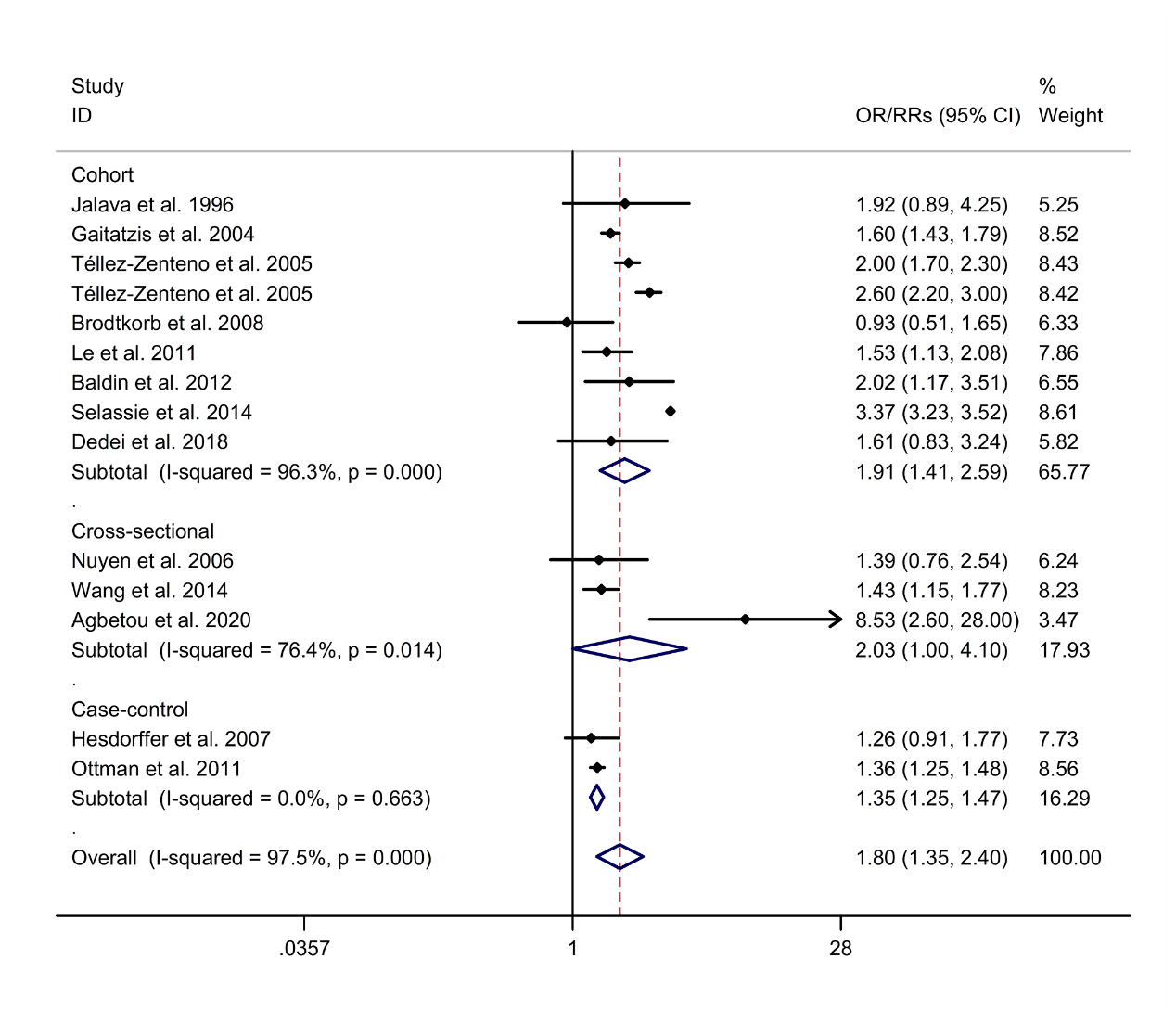


Supplementary figure 2. Subgroup analysis for association between epilepsy and risk of migraine in different study types. Abbreviations: CI, confidence interval; OR, odds ratio; RR, relative risk.


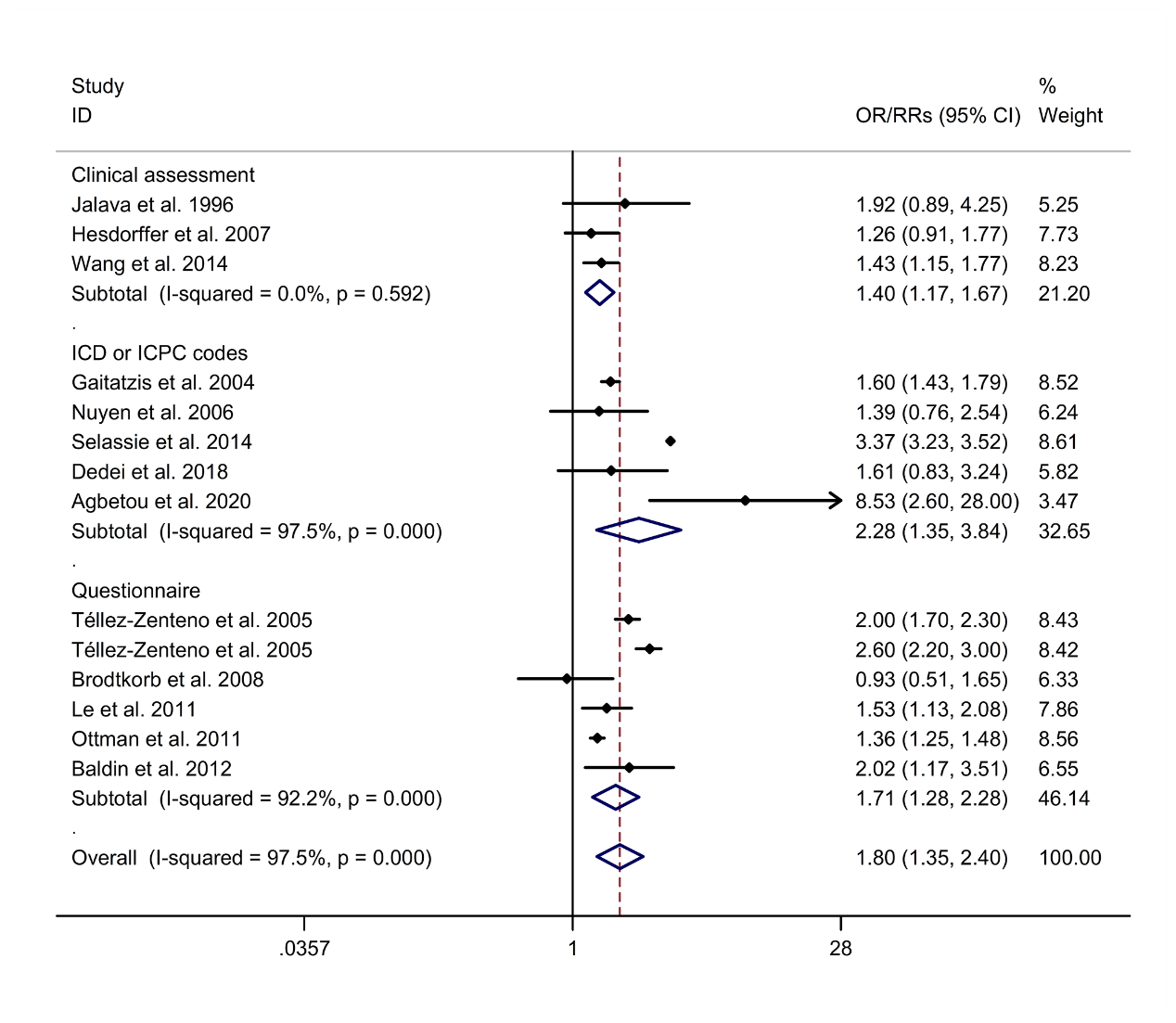


Supplementary figure 3. Subgroup analysis for association between epilepsy and risk of migraine diagnosed with different methods. Abbreviations: CI, confidence interval; OR, odds ratio; RR, relative risk.


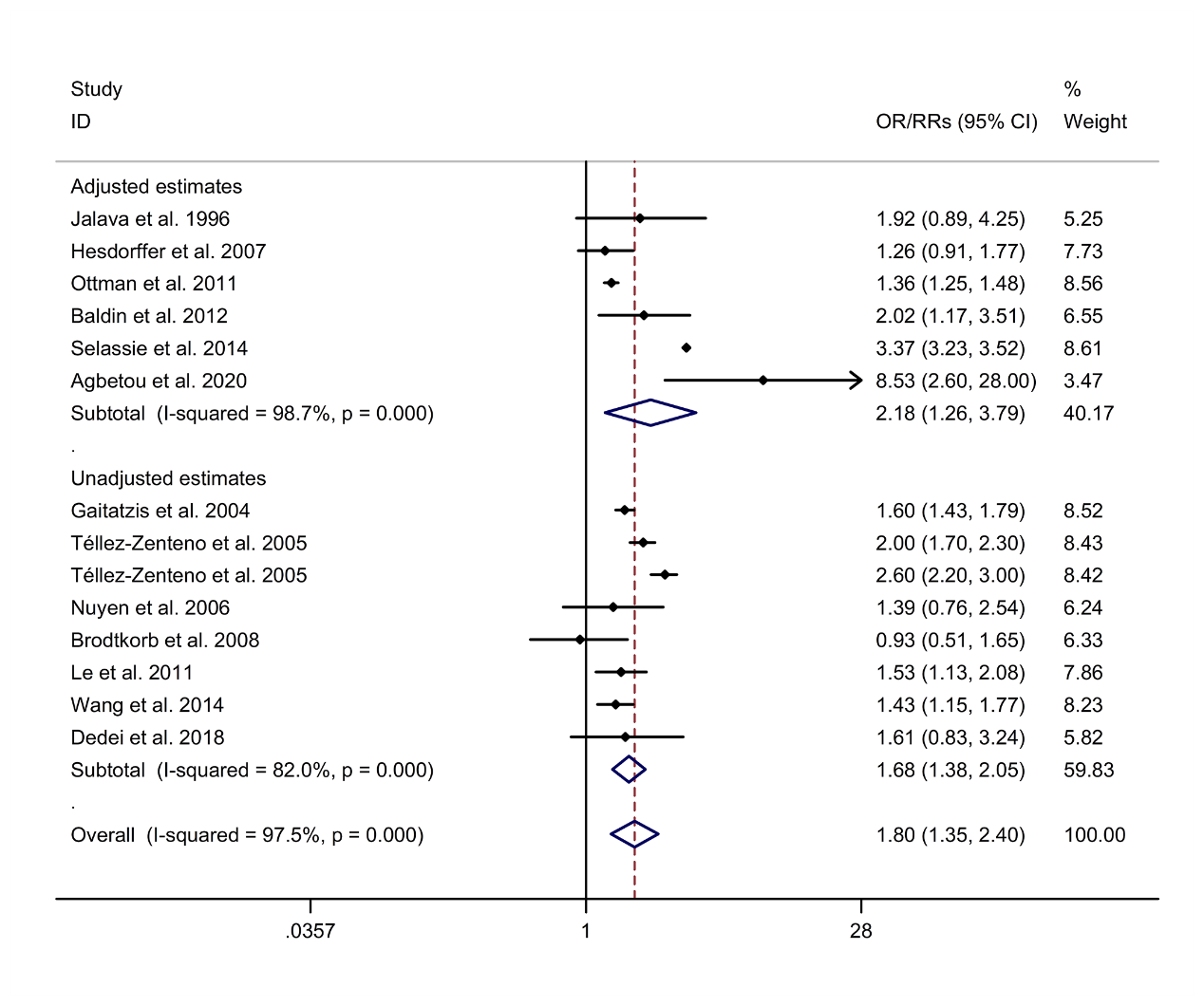


Supplementary figure 4. Subgroup analysis for association between epilepsy and risk of migraine with adjusted or unadjusted effect estimates. Abbreviations: CI, confidence interval; OR, odds ratio; RR, relative risk.


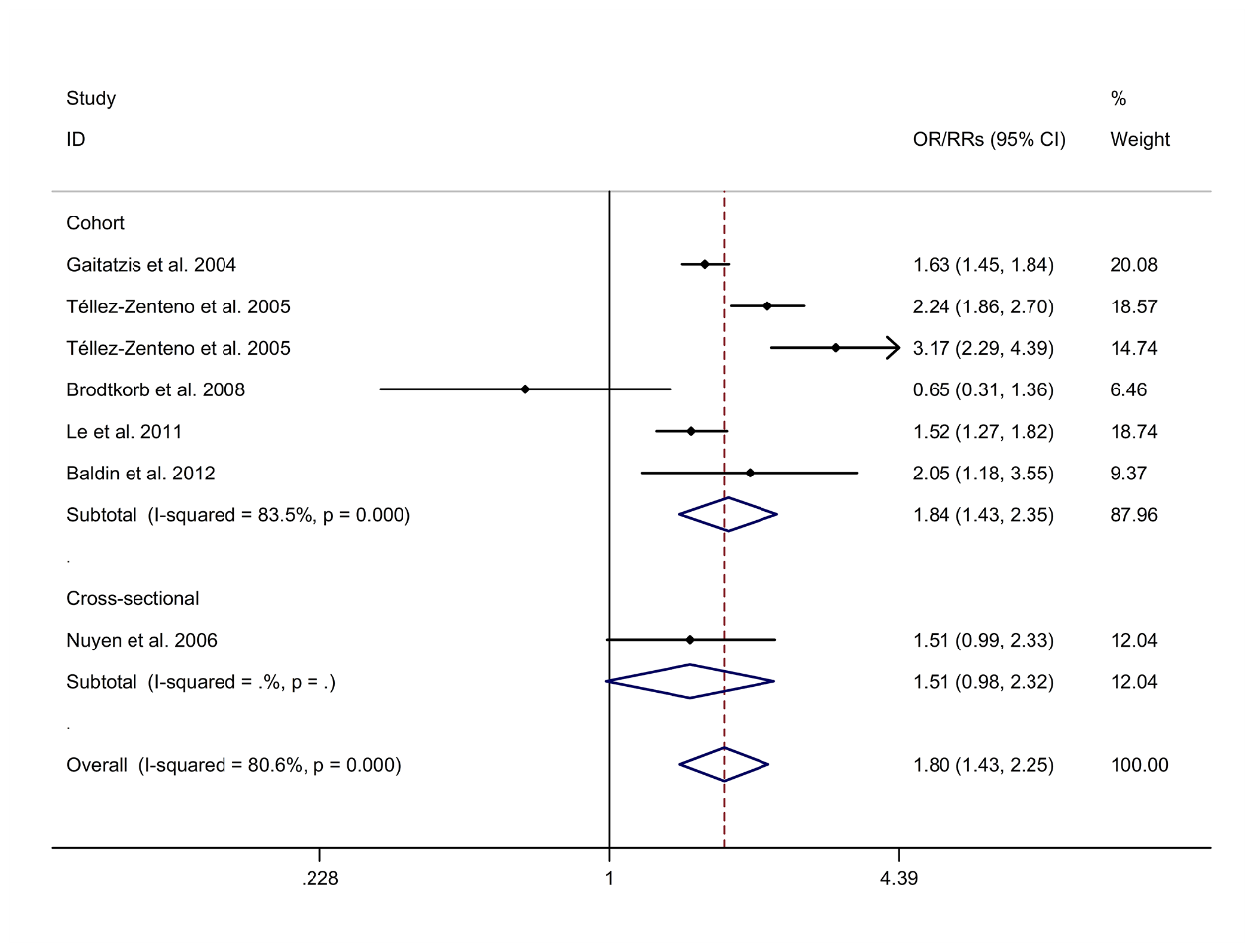


Supplementary figure 5. Subgroup analysis for association between migraine and risk of epilepsy in different study types. Abbreviations: CI, confidence interval; OR, odds ratio; RR, relative risk.


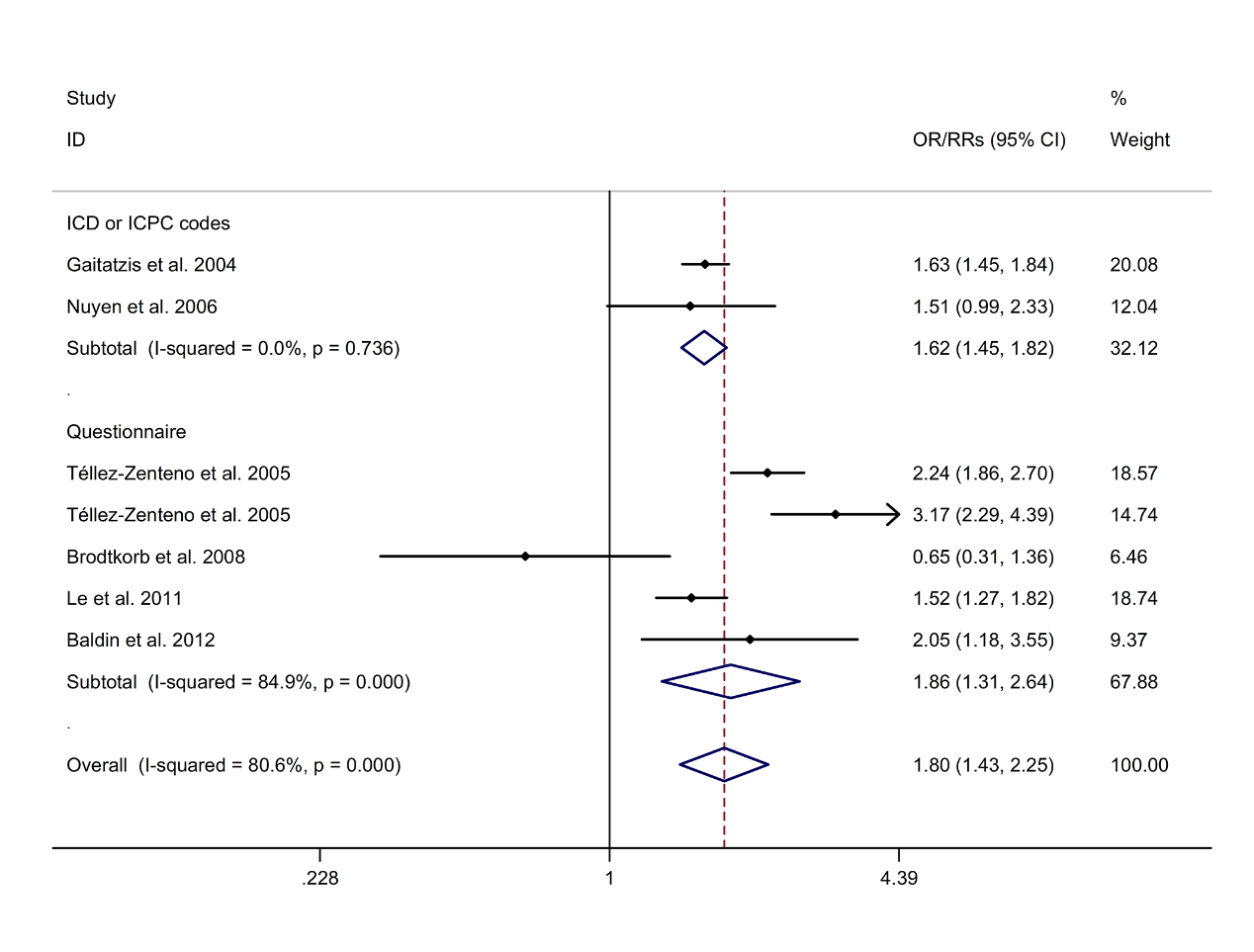


Supplementary figure 6. Subgroup analysis for association between migraine and risk of epilepsy diagnosed with different methods. Abbreviations: CI, confidence interval; OR, odds ratio; RR, relative risk.


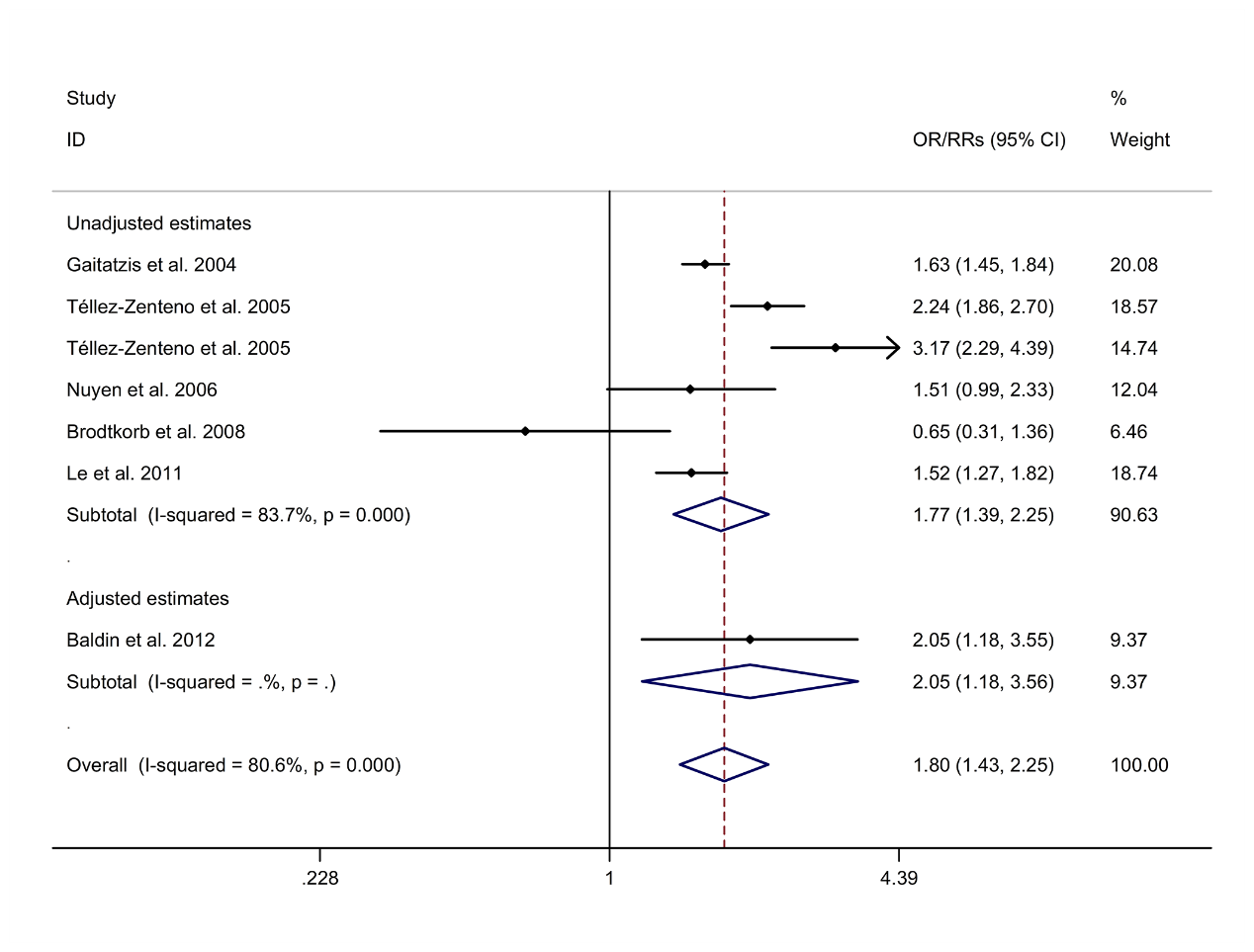


Supplementary figure 7. Subgroup analysis for association between migraine and risk of epilepsy with adjusted or unadjusted effect estimates. Abbreviations: CI, confidence interval; OR, odds ratio; RR, relative risk.
